# Minor psychiatric disorders among Brazilian ragpickers: a cross-sectional study

**DOI:** 10.1186/1476-069X-5-17

**Published:** 2006-05-30

**Authors:** Marcelo Cozzensa da Silva, Anaclaudia Gastal Fassa, David Kriebel

**Affiliations:** 1 School of Medicine, Department of Social Medicine, Post-graduate Program in Epidemiology, Federal University of Pelotas, Brazil. Av. Duque de Caxias, 250, Third floor, Pelotas, Rio Grande do Sul 96030-002, Brazil; 2 Department of Work Environment, One University Avenue, Lowell, Massachusetts, 01854, USA

## Abstract

**Background:**

Ragpickers are informal workers who collect recyclable materials to earn a small wage. Their life and working conditions are extremely difficult. We examined minor psychiatric disorders (MPD) among a cohort of ragpickers in Pelotas, a city in southern Brazil.

**Methods:**

Ragpickers were matched by sex, age, and years of schooling with a sample of non-ragpickers from the same poor neighborhoods. The cross-sectional study gathered data by interview on 990 individuals in 2004. MPD were assessed using a standard self-reporting questionnaire, the SRQ-20.

**Results:**

The prevalence of MPD among ragpickers was 44.7%, higher than reported by neighborhood controls (33.6%; p < 0.001). MPD were more common among females, those of lower economic level, smokers and alcoholics. Among occupational characteristics, MPD prevalence was associated with frequent static postures, low job satisfaction and recent work accidents.

**Conclusion:**

Ragpickers more frequently report MPD than other poor workers living in the same neighborhoods, with many of the same life conditions. Improving the work lives of these precarious workers should address not only the physical hazards of their jobs but their mental and emotional health as well.

## Background

Mental disorders rank almost as high as cardiovascular diseases in the total global burden of diseases (9.7% versus 10.5%, respectively)[1]. It is estimated by WHO[2] that depression will be the single most important cause of disability by the year 2020 in the developing world.

Minor psychiatric disorders have long been associated with work, and in 1991 were the second leading cause of lost work time in the United States[3]. Studies have demonstrated that socioeconomic deprivation, resulting from unemployment is associated with various psychological disorders[4,5].

A new study by the International Labor Organization Office (ILO) has reported that 2.8 billion people in the world were employed in 2003. Of these, nearly 1.4 billion were living on less than the equivalent of US$2 a day and some 550 million were living under the US$1 a day poverty line[6]. In Brazil, the official estimate for the number of unemployed people in December 2002 was 2.1 million people[7], although non-official sources say the real number may be three times higher. A large number of these unemployed in Brazil have found an alternative to survive by working in garbage. The ragpickers ("catadores de material reciclável") survive from the collection, separation, classification and sale of municipal solid waste.

**Figure 1:**
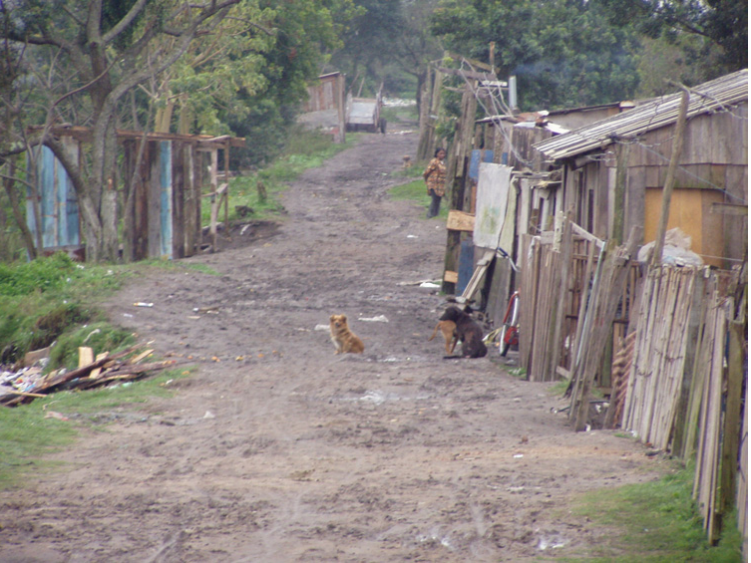
Ragpickers live in poor quality houses, often with no running water or electricity.

**Figure 2:**
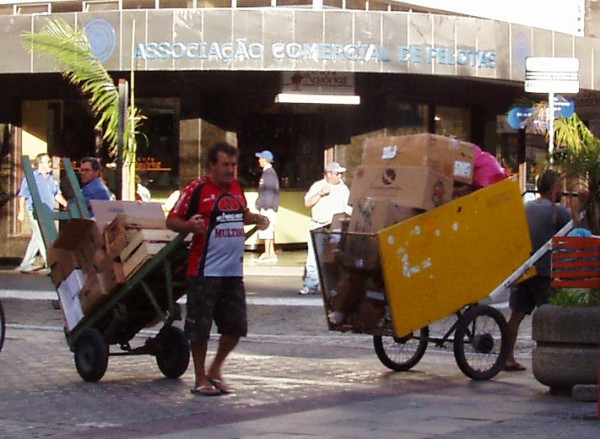
Ragpickers and their pushcarts are a common sight on the streets of Pelotas city.

It is not known how many people work as ragpickers in Brazil, but a recent study estimated 500,000 in 2003, comprising both adults and children[8]. The majority of these workers have incomes less than twice the level defined by the Brazilian government as a minimum living wage, which comes to about US$173/month. They often live near dumps or in the low income areas of cities (Figure 1), and collect recyclable materials and food at dumpsites, riverbanks, street corners and residential areas[8]. (Figure 2)

The ragpickers work in hazardous conditions. When moving around in the garbage, searching for materials that can be resold, the ragpickers are exposed to a wide range of health and safety hazards; ranging from infectious agents and toxic chemicals, the handling of sharp or broken materials and serious musculoskeletal strain[9,10]. The ergonomic hazards include those experienced in other manual materials handling jobs, including static or awkward postures, physical efforts, such as, lifting, loading or pushing heavy weights, and repetitive movements [11-14].

Although more than 60% of the workforce in Brazil is in the informal sector, there are few studies on work and health of this population segment[15], and ragpickers are no exception. The studies in the literature are few and only descriptive. One reason for this lack of research is the considerable logistical challenge of conducting epidemiologic studies of people without regular places of work or residence. The objective of this article was to examine the association of minor psychiatric disorders with the conditions of life and work of the ragpickers and compare these to non-ragpickers from the same city.

## Methods

### Survey populations and questionnaire

Pelotas is a city located in the state of Rio Grande do Sul, southern Brazil. It has a population of 338,000 inhabitants, the majority of them (94%) living in urban areas. Almost 25% of the population earns less than twice the national minimum wage (US$ 138.50/month in 2002) with 14.6% receiving more than ten times the minimum wage.

Eighty five percent of the city population is of white race with most of the rest of mixed African and European descent. Until the past decade, the city was known for a heavy concentration of food production industries: first meat packing and later canning and processing of fruits and vegetables. This industry has been in serious decline recently, and the number of unemployed has increased substantially.

A cross-sectional study was carried out among ragpickers and neighborhood matched referents from March to July 2004. In order to identify the ragpickers, field researchers went to the main points of sale of recycled materials and gathered names and addresses of everyone that came to sell materials. Ragpickers were also identified in cooperatives of recycled materials, by key-informants in poor neighborhoods, and by ragpickers who led us to others doing the same work in the neighborhoods where they lived. After developed a list of ragpickers, the interviewers visited their places of residence. Only people 18 years or older were asked to participate. The interviewers excluded all individuals with mental disorders that limited their ability to answer the questionnaire.

The referent group was composed of other workers that lived in the same neighborhoods as the ragpickers. To locate referents, interviewers went to neighboring houses, starting from those immediately adjacent to each subject's home, in order to locate a suitable non-ragpicker to interview. These people were matched by gender, age (± 5 years old), and years of schooling (± 1 year) to the neighboring ragpicker. Potential referents were excluded if they were unemployed, retired or out of work because of any health problem.

Trained interviewers used a structured questionnaire to gather data on occupational, socio-demographic, economic, behavioral, health factors and work. The interview lasted approximately 40 minutes. Five percent of interviews were repeated by the principal investigator (M.S.) as a check on the quality of data collection.

The prevalence of minor psychiatric disorders was assessed using the Self-Reporting Questionnaire – SRQ-20[16]. This instrument was designed by Harding et al. for the World Health Organization (WHO) to study common mental disorders in primary health care, and it is applicable in different cultural settings, especially in developing countries[17]. This instrument is used to screen minor psychiatric disorders like depression and anxiety and is composed of 20 "yes/no" questions: four on physical symptoms and sixteen on psycho-emotional disturbance. These 20 questions are listed in Table 1. An advantage of using SRQ-20 was that it has been validated in Brazilian urban settings [18-20] and in other studies of workers' health, showing high sensitivity and specificity[21]. For example, Mari used this instrument in a study of 875 psychiatric patients in São Paulo, 260 of whom were then interviewed by a psychiatrist. The best values of sensitivity and specificity were obtained using different cutoff points for men and women. For men, the authors found the instrument had a sensitivity of 89% and specificity of 81% when a score of six or more positive responses was taken as indicating the presence of MPD. For women, the sensitivity was 86% and the specificity 77% when the cutoff was eight or more positive responses [20]. We used these same gender-specific cutoffs. The SRQ-20 is available in Portuguese and has been used in a number of studies of worker's health in Brazil [22-28], thus allowing for a better comparison of our results to those for other occupational categories. The SRQ-20 has been shown to be a cost-effective way to evaluate mental health in developing countries. It has high validity and well over ten years' history of application in at least 20 countries[29].

**Table 1:** Self-Reporting Questionnaire (SRQ-20) 20 items [29]

1. Do you often have headaches?	Yes/No
2. Is your appetite poor?	Yes/No
3. Do you sleep badly?	Yes/No
4. Are you easily frightened?	Yes/No
5. Do your hands shake?	Yes/No
6. Do you feel nervous, tense, or worried?	Yes/No
7. Is your digestion poor?	Yes/No
8. Do you have trouble thinking clearly?	Yes/No
9. Do you feel unhappy?	Yes/No
10. Do you cry more than usual?	Yes/No
11. Do you find it difficult to enjoy your daily activities?	Yes/No
12. Do you find it difficult to make decisions?	Yes/No
13. Is your daily work suffering?	Yes/No
14. Are you unable to play a useful part in life?	Yes/No
15. Have you lost interest in things?	Yes/No
16. Do you feel that you are a worthless person?	Yes/No
17. Has the thought of ending your life been on your mind?	Yes/No
18. Do you feel tired all the time?	Yes/No
19. Do you have uncomfortable feelings in your stomach?	Yes/No
20. Are you easily tired?	Yes/No

In this paper, we also investigated the degree to which ragpickers' exposures and outcomes varied according to several demographic and personal factors. These were: age, gender, skin color (white/non white), marital status (living with or without a partner), monthly income (categories of multiples of the official minimum wage), years of schooling, smoking (never smokers; ex-smokers; current smokers), alcoholism (using the standard CAGE instrument)[30] and economic level. This last characteristic was assessed using a standard Brazilian scale, the ABEP [31] (Table 2). We used this standardized scale for socioeconomic position in order to compare our results with the results of a recent study of MPD in the general Pelotas city population [32]. Using a series of 10 questions on household items owned by the respondent (radio, television, refrigerator, vacuum, wash machine, and so on) and the level of education of the head of the family, a score is calculated and respondents are placed in one of five levels from A (highest) to E (lowest) economic level. A limitation of this instrument is that, nowadays, people with low income have greater access to electric and electronic equipment, and because of this, "poor people" are classified into higher categories of the ABEP. While this may introduce some inaccuracy into the social class ratings we have used, it is not likely to be a differential bias between ragpickers and their neighborhood non-ragpicker referents.

**Table 2:** ABEP scale of socioeconomic position. The first table indicates how different types and numbers of household possessions are counted to yield a combined score of economic status [31]. The second table indicates the weight given to head of household education level, and the third table translates the total household score into a class. There were no ragpickers in class A.

	Number of items at home				
	0	1	2	3	4 or more
Color TV	0	2	3	4	5
Radio	0	1	2	3	4
Bathroom	0	2	3	4	4
Car	0	2	4	5	5
Maid	0	2	4	4	4
Vacuum cleaner	0	1	1	1	1
Washing machine	0	1	1	1	1
VCR or DVD	0	2	2	2	2
Refrigerator	0	2	2	2	2
Freezer	0	1	1	1	1

Level of education of the head of the family					
				
Schooling	Score				
				
0 to 4 years of schooling	0				
5 to 7 years of schooling	1				
8 to 11 years of schooling	2				
12 to incomplete college	3				
Graduate	5				
				
Total score and its equivalent class					
				
Score	Class				
				
30 – 34	A1				
25 – 29	A2				
21 – 24	B1				
17 – 20	B2				
11 – 16	C				
6 – 10	D				
0 – 5	E				

Physical hazards were also evaluated by the questionnaire. These included static postures at work ("do you stay in the same position for a long time at work?")[33], work accidents ("did you have any work accident in the last 12 months?), and job satisfaction ("are you satisfied with your work?").

Musculoskeletal pain was assessed by the Standardized Nordic Questionnaire[34], and the symptoms were divided into three regions: lower back, the lower extremities (upper leg, knee, lower leg and ankle) and upper extremities (neck, shoulder, elbow and wrist) [35].

### Statistical analysis

The data were entered into a computer database twice by different technicians and compared. Discrepancies were resolved by reference to the original survey. Analyses were conducted using Stata 8.0. Prevalences and prevalence ratios were calculated to compare exposures and outcomes among groups. Prevalence ratios were calculated using Poisson regression in order to investigate potential confounding and effect modification[36]. Multivariate modeling to identify factors associated with MPD used the approach of Victora et al[37], in which the effects of demographic variables (age, sex, gender, marital status, education) were investigated first. Smoking, alcoholism and an indicator variable for being a ragpicker were added in a second stage, and finally work hazards and job satisfaction were added in a third stage. After this, musculoskeletal disorders and work accidents were added to the model. This four-step "hierarchical" method helps the researchers to understand when a proximal factor related to work (lifting for example) may be acting as a mediator for a more distal social factor like education. Multivariate modeling began by adding Level One variables one at a time, to identify important predictors. Then, jobs/exposure variables were added one at a time. Two-way interactions between first and second level variables were evaluated using product terms.

## Results

We interviewed 455 of the 546 ragpickers initially identified (83.3%). The 91 who were not interviewed were those whose residence could not be located. This occurred when a ragpicker reported a non-existent address at first contact, the interviewer failed to find the address through lack of street signs or names, or because some had moved away between first contact and household visit. Neighborhood referents were successfully identified and interviewed for each of the 455 ragpickers, bringing the study population to 990. There were 80 household members residing with the matched referents that were excluded from analyses in order to match one ragpicker to one non-ragpicker. There were many more non-ragpickers with high levels of education (4.6% versus 3.1% with more than 8 years of schooling, respectively). Because education was a potentially important modifier of job characteristics, we chose to study only those respondents with 8 or less years of schooling. Our final sample was 881 individuals (441 ragpickers and 440 non-ragpickers).

In our study, the ragpickers reported considerably poorer living conditions than their neighbors with other occupations (Table 3). For example, the majority (54.0%) of ragpickers lived in poor quality houses built of plastic, metal or wood, while only a quarter of their matched neighbors (25.0%) lived in such houses. Fifteen percent of ragpickers had no running water, but only 4.8% of non-ragpickers (p < 0.001). Nearly twice as many ragpickers as non-ragpickers had no electricity (11.0% versus 5.7%; p = 0.003). Eighteen percent of ragpickers, but only 3.0% of referents reported having no toilet (p < 0.001). On average, there were 4.7 residents in a ragpicker's home, and only 3.9 in a non-ragpicker's.

**Table 3:** Demographic characteristics and living conditions of ragpickers and non-ragpickers (n = 881).

**VARIABLE**	**RAGPICKERS %**	**NON-RAGPICKERS %**	**P value**
Age (Years)*			0.60
18 to 29 years old	31.3	28.0	
30 to 39 years old	26.8	28.6	
40 to 49 years old	24.0	25.5	
50 to 59 years old	11.1	12.7	
60 to 69 years old	6.8	5.2	
			
Gender*			0.90
Male	62.6	62.9	
Female	37.4	37.1	
			
**Skin color**			<0.001
White	53.1	67.0	
Non white	46.9	33.0	
			
Elementary education (Years)*			0.01
< 1	23.1	15.2	
1 to 4 years	45.1	50.0	
5 to 8 years	31.8	34.8	
			
**Running water at home**			<0.001
No	15.2	5.0	
Yes	84.8	95.0	
			
**Electricity at home**			0.01
No	10.4	5.9	
Yes	89.6	94.1	
			
**Toilet at home**			<0.001
No	18.6	3.2	
Yes	81.4	96.8	
			
**House build materials**			<0.001
Bricks	46.0	74.5	
Poor wood, plastic, cardboard	54.0	25.5	

* matching variable; differences between ragpickers and non-ragpickers are those that remained after matching, and after dropping 35 subjects with > 8 years of schooling

As noted, there were no ragpickers in the highest economic level, ABEP category A, while 21.9% were in the intermediate categories B or C, and 78.1% in the lowest economic level categories D or E. For comparison, data from a recent survey of Pelotas general population found 5.6%, 62.8% and 31.6% in categories A, B or C, and D or E, respectively[32].

Both ragpickers and referents had a mean age of 38 years, and were 63.0% male (matching variables). Despite matching to within one year on schooling, ragpickers were still more poorly educated then their neighbors. Most strikingly, 23.1% of ragpickers, but only 15.2% of non-ragpickers had not completed one year of schooling (p = 0.05). This discrepancy would have been much larger without matching; a non-ragpicker with one year of schooling was often matched to a ragpicker with no schooling. There were large racial differences between groups: 46.9% of ragpickers were non-white compared to 33.0% of their neighbors (p < 0.001). The average monthly incomes of ragpickers and non-ragpickers were respectively US$80.10 and 182.30. Almost all (94.6%) of ragpickers reported less than twice the Brazilian basic wage (equivalent US$86.70/month) compared to 64.7% of non-ragpickers. Domestic work (28.0%), day laborers (33.4%) retail sales (14.6%) and construction (13.4%) were the most frequently reported occupations of the non-ragpickers neighborhood referents.

More than 90.0% of ragpickers reported that their work was highly repetitive, compared to 65.5% of their neighbors. Frequent lifting, static postures and vibration were all considerably more prevalent in the work of ragpickers than in the comparison group. Frequent squatting was almost twice as common among ragpickers as among non-ragpickers (43.1% vs. 22.1%; p < 0.001).

### Minor psychiatric disorders (MPD)

The prevalence of MPD in ragpickers (44.7%) and non-ragpickers (33.6%) was different (p = 0.001). When the non-ragpickers were sub-divided by occupation, the prevalence of MPD among domestic workers, day laborers, retail and construction workers were 39.0%, 36.3%, 35.9%, 23.7% respectively. Thus, all other occupations reported MPD prevalences less than among ragpickers.

Univariate models identified female gender, and working as a ragpicker as being associated with the studied outcome (Table 4). People from economic levels C, D and E showed higher prevalences of MPD than level B. Years of schooling was inversely associated with the outcome. MPD prevalence was not associated with age, marital status or skin color (data not shown). Current smokers had 30% more risk for MPD than non-smokers, and there was a similar prevalence ratio comparing alcoholics to non-alcoholics. Workers who reported frequent static postures, a recent work accident, or low job satisfaction were also more likely to report MPD (Table 4). Low back pain (LBP), lower extremity pain (LEP) and upper extremity pain (UEP) were each associated with a 70 to 80% higher prevalence of MPD compared to the absence of these symptoms (data not shown).

**Table 4:** Prevalence, prevalence ratios and confidence intervals for minor psychiatric diseases by socio-demographic, behavioral, and work characteristics for the sample (n = 879)^§^.

**Variables**	**Prevalence (%)**	**PR (CI 95%)**	**P value**
Gender*			0.001
Male	35.1	1.0	
Female	45.9	1.3 (1.1 – 1.5)	
			
**Economic Level (ABEP)**			0.01^&^
Level B (Highest)**	26.3	1.0	
Level C	31.8	1.2 (0.5 – 2.5)	
Level D	39.9	1.5 (0.7 – 3.1)	
Level E (Lowest)	43.4	1.6 (0.8 – 3.4)	
			
**Schooling (Years)**			0.02^&^
<1	46.2	1.0	
1 a 4 years	39.3	0.9 (0.7 – 1.0)	
5 a 8 years	34.8	0.8 (0.6 – 1.0)	
			
**Smoking Status**			0.01^&^
Never	33.9	1.0	
Ex-	36.7	1.1 (0.8 – 1.4)	
Current	43.3	1.3 (1.1 – 1.5)	
			
**Alcoholism**			<0.001
CAGE Negative	36.1	1.0	
CAGE Positive	58.3	1.3 (1.2 – 1.4)	
			
**Ragpickers**			0.001
No	33.6	1.0	
Yes	44.7	1.3 (1.1 – 1.6)	
			
**Static Posture**			0.001
No	30.6	1.0	
Yes	42.4	1.4 (1.1 – 1.7)	
			
**Work accidents**			<0.001
No	36.1	1.0	
Yes	58.3	1.6 (1.4 – 1.9)	
			
**Job satisfaction**			<0.001
Yes	36.1	1.0	
No	58.3	1.6 (1.4 – 1.9)	

^§ ^Those with more than 8 years of schooling were excluded.

^&^p for trend.

* Cut-points defining MPD: Males ≥ 6 Females ≥ 8. See text for details.

**There were no participants from economic level A.

MPD prevalence was not associated with body mass index. For example, the mean values of BMI for men above and below the SRQ-20 cutoff were 40.6 and 41.2, respectively (p = 0.40). For women, the difference was 39.8 versus 41.1(p = 0.30). Body mass index was not considered in further analyses.

These univariate associations with MPD prevalence were then evaluated for potential confounding and effect modification in Poisson regression models. First we analyzed the demographic and behavior variables. Women consistently reported a higher prevalence of MPD than men, and this association was not diminished after controlling for economic level and schooling. Lower economic level showed an inverse trend with MPD, such that those in the lowest level, E, were at the highest risk (PR = 1.6). When adjusted for gender and economic level, schooling became only weakly associated with MPD (p = 0.4). Alcoholism (PR 1.3, 95%CI 1.2–1.4) was significantly associated with MPD. There was a borderline statistical significance for the association between smoking and MPD (p = 0.09) when a 3 level smoking variable was included. The current smokers' prevalence ratio was 1.3 (95%CI 1.0–1.5) compared to non-smokers (Table 5).

**Table 5:** Poisson regression models estimating prevalence ratios and 95% confidence intervals for minor psychiatric diseases, by socio-demographic, behavioral, and work characteristics*.

**VARIABLES**	PR Adjusted** PR (95% CI)	**P value**
**Economic Level**^**1**^		0.008
Level B	1.0	
Level C	1.2 (0.5 – 2.5)	
Level D	1.5 (0.7 – 3.1)	
Level E	1.6 (0.8 – 3.4)	
		
**Smoking Status**^**2**^		0.09
Never	1.0	
Ex-	1.1 (0.9 – 1.5)	
Current	1.3 (1.0 – 1.5)	
		
**Alcoholism**^**2**^		<0.001
CAGE Negative	1.0	
CAGE Positive	1.3 (1.2 – 1.5)	
		
**Ragpickers**^**3**^		0.01
No	1.0	
Yes	1.3 (1.0 – 1.5)	
		
**Static Posture**^**4**^		0.02
No	1.0	
Yes	1.3 (1.1 – 1.6)	
		
**Job satisfaction**^**4**^		<0.001
Yes	1.0	
No	1.5 (1.3 – 1.8)	
		
**Work accidents**^**4**^		<0.001
No	1.0	
Yes	1.4 (1.2 – 1.7)	

* Only those variables shown to have univariate association with MPD are shown (gender, age and schooling were associated with MPD but because they were matching variables, they could not confound other associations, and so they are not shown here).

** PR adjusted for all variables in the same level and the previous levels.

^1^First level. ^2^Second level. ^3^Third level. ^4^Fourth level.

After characterizing these demographic and behavioral characteristics of symptom prevalence, we then investigated the possibility that the lack of strong differences in symptom prevalence between ragpickers and non-ragpickers might be explained by confounding by one or more of these characteristics. Because we had matched closely on age and gender, these could not confound the ragpicker-symptom associations. The sample was also matched on schooling, but as noted above, there was a substantial residual difference in schooling between ragpickers and non-ragpickers. Despite this, models with and without schooling showed no differences in other factors, and so models with schooling are not discussed further.

Table 5 shows a single model with all potential confounders included, and the ragpicker/non-ragpicker effect is only slightly reduced. Similarly, models with each of the variables in Table 5, added one at a time, did not change the ragpicker-MPD association materially.

We therefore investigated the associations between occupation (ragpicker/non-ragpicker), static posture, job satisfaction and work accidents with MPD. We investigated confounding and effect modification of these associations by economic level. Being a ragpicker was associated with a 20% higher prevalence of MPD. Static posture, job satisfaction and work accidents were associated with increases of 30% (95% CI= 1.1–1.6), 50% (95% CI = 1.3–1.8) and 40% (95% CI= 1.2–1.7) respectively in the symptom prevalence (Table 5).

## Discussion

Work in the informal sector is increasing in most countries, and involves an estimated 60% of the workforce in Brazil, a large developing nation. Understanding the health, environmental, social and economic implications of this trend presents important challenges to public health researchers and administrators. This paper examined the risk factors for MPD among ragpickers and their non-ragpicker neighbors in Pelotas, Brazil. The problem of MPD has been described in many occupations in Brazil[22,24,27], but rarely among those in the informal sector. This study is one of the first to use a quantitative analytic approach to studying the ragpickers' lives and working and health conditions, especially MPD.

Before discussing the results, it is important to address certain methodological aspects of the study. Data were collected using a standardized instrument, by a trained team, and in an identical fashion in both groups, thus contributing to the internal validity of the study.

Cross-sectional studies of working populations are often biased towards underestimation of effects through healthy worker selection[38]. Selection out of the work force of those with MPD is particularly problematic in cross-sectional studies. In our study, we believe that this bias may not have been strong because the people that perform this kind of job are those who are unemployed, and have very few alternatives but to continue this work, regardless of their health status. The most important thing for them is to perform their work in order to earn some money to survive. Ambiguous temporal ordering is another inherent problem in cross-sectional studies. It is possible that some of the association between being a ragpicker and MPD could be due to reverse causality, in which those suffering from MPD find work as ragpickers. We believe, however, that the more important explanation is that stressful life and work conditions of ragpickers increase their risk of MPD. We note, for example, that the ragpickers were relatively young: more than 75% were between 18 and 49 years; 66% reported that they had chosen this work because they had lost a job; and that very few of them reported pre-existing mental pathologies (survey data on these variables not shown).

Minor psychiatric disorders are not the only, or perhaps even the most important morbidity that ragpickers face[9]. Rather, we wanted to show that careful application of standard epidemiologic methods enabled us to systematically evaluate a range of problems faced by workers in the informal sector. A second paper reports our findings on musculoskeletal pain in this population [35].

The reported prevalence of MPD (44.7%) was higher than that reported for formal sector workers in other studies using the same instrument. For example, prevalences of about 12% have been reported for studies in bus drivers[24,27], 23.9% among managers in a state-owned company[39] and 24.6% and 19.1% among dentists and college professors respectively[23].

The socioeconomic factors that condition the life of these poor people are likely to have a close connection to MPD[32]. In our study, the people included in the lowest economic levels, ABEP classes D and E, had prevalence ratios of 1.5 and 1.6 respectively when compared to those in Level B. Low income groups are more vulnerable to MPD, irrespective of the overall state of development of the society in which they live[5].

Minor psychiatric disorders were considerably more common among women than men in our study. This pattern has been reported in many other populations[32,40-42]. This difference in prevalence occurred despite the common practice of using a higher cutoff for identifying MPD in women (8) than in men (6)[28].

An earlier study of ragpickers in Brazil found a high prevalence of anxiety among this population[43]. Informal workers, especially ragpickers, have lower status than formal workers, lack security of employment and have less control over their working conditions[26]. The work stresses that they face include: inherent dangers of their work sites (dumps, riverbanks, roadsides), their lack of personal protective equipment, the risk of traffic accidents because they often work amidst heavy traffic at intense traffic hours, irregular hours of work including at night, social isolation and discrimination by society, and considerable financial insecurity. It is reasonable to assume that all of these can have negative impacts on their mental health[9].

The work of ragpickers involves frequent static postures[9], and this has been previously linked with musculoskeletal pain[13,44,45]. These in turn may lead to depression and anxiety[46]. Monotonous work has been associated with psychological distress[47].

Job satisfaction and work accidents were associated with MPD in our study. Similarly, Jurado et al[41] found that low job satisfaction increased the risk of depressive symptoms among school teachers. It is not hard to imagine that a recent accident could increase anxiety and depression among workers who are barely subsisting, and have no safety net if they cannot work. Despite this, caution is needed in interpreting cross-sectional studies causally; we cannot be confident that the low job satisfaction and work accidents preceded MPD.

## Conclusion

Unemployment has been acknowledged as an important determinant of MPD in both developed and developing countries. Research concerned with the relationship between employment and health has often focused on the experience of relatively affluent countries, where several forms of welfare provide at least minimal protections for the unemployed. But in Brazil and many other developing countries, a large fraction of the population works outside the formal labor market and have no social safety net. Over the past 20 years in Brazil, the number of people who work in the collection of recyclable materials (ragpickers) has increased dramatically. We found that the prevalence of MPD was higher in a sample of ragpickers than among neighbors who worked in more traditional manual labor, such as domestic work and construction. More attention should be paid to these workers that play an increasingly important role in the Brazilian economy and its environmental management. Educational programs should be introduced, adequate job training, appropriate awareness of the risks of the job and of the health problems that may arise. They should have access to personal protective equipment, materials handling devices, and safe means of transportation.

Perhaps most importantly, means should be found to bring these workers into the formal economy; ensuring them a basic wage, job security, and the social status that comes with a "real" job. Some Brazilian cities, including Pelotas, have begun to support the establishment of ragpicker cooperatives to help these workers collectively transform this work into a decent and respectable occupation [48,49]. More than an occupational health issue, the ultimate goal should be for ragpickers to escape their current marginality and to obtain respect and dignity. One small step in that direction was the recent inclusion of the occupation of "*ragpicker*" in the new Brazilian Occupation classification in 2002.

Understanding the causes of MPD in different societies and jobs requires an understanding of the different socioeconomic circumstances around the world. Working outside the protection of employment legislation is very common in many poorer countries. It is an aspect of socioeconomic inequalities that has a particular meaning in a society like Brazil and that may have important consequences for mental health[26].

## Competing interests

The author(s) declare that they have no competing interests.

## Authors' contributions

MCS participated in the design of the study, supervised data collection, performed the data analyses and drafted the manuscript; AGF participated in the design of the study and helped to draft the manuscript, DK helped in data analyses and to draft the manuscript. All authors read and approved the final manuscript.
